# A Pilot Study Using the Life Space Assessment Among Community-Dwelling Older Adults in Ireland

**DOI:** 10.1093/geroni/igaa057.2336

**Published:** 2020-12-16

**Authors:** Tadhg Stapleton

**Affiliations:** Trinity College Dublin, Dublin, Dublin, Ireland

## Abstract

Forty older adults (27, 67.5% female), mean age 74.3 years (range 65- 89), current drivers 31 (77.5%), 23 (57.5%) rural dwellers and 17 (42.5%) urban dwellers participated in the survey. Median LSA score was 74 (range 27-102). All participants were readily accessing life spaces within and immediately outside their own home. Over half (n=23, 57.5%) accessed spaces in their neighbourhood on a daily basis. Decreased frequency of access to spaces outside of local neighbourhood and town was noted. No significant difference in LSA scores between genders (P=0.549), current driving status (P=0.235), but urban dwellers had significantly higher LSA scores than rural dwellers (P=0.024). Spearman correlations found statistically significant negative correlation between age and LSA scores (rho= -0.445, P=0.004), and significant positive correlation between LSA and Euroqol (EQ) VAS scores (rho=0.405, P=0.010). Findings are limited by the small sample size but highlight decreasing frequency of wider community participation with increasing age.

